# A Fast ML-Based Single-Step Localization Method Using EM Algorithm Based on Time Delay and Doppler Shift for a Far-Field Scenario

**DOI:** 10.3390/s18124139

**Published:** 2018-11-26

**Authors:** Tianzhu Qin, Lin Li, Bin Ba, Daming Wang

**Affiliations:** National Digital Switching System Engineering and Technology Research Center, Zhengzhou 450002, China; skypillar@outlook.com (T.Q.); xidianbabin@163.com (B.B.); wdm_wangdaming@163.com (D.W.)

**Keywords:** direct position determination (DPD), maximum likelihood (ML), expectation maximization (EM), Doppler shift, Laplace approximation, Cramér–Rao bound (CRB)

## Abstract

This study discusses the localization problem based on time delay and Doppler shift for a far-field scenario. The conventional location methods employ two steps that first extract intermediate parameters from the received signals and then determine the source position from the measured parameters. As opposed to the traditional two-step methods, the direct position determination (DPD) methods accomplish the localization in a single step without computing intermediate parameters. However, the DPD cost function often remains non-convex, thereby it will cost a high amount of computational resources to find the estimated position through traversal search. Weiss proposed a DPD estimator to mitigate the computational complexity via eigenvalue decomposition. Unfortunately, when the computational resources are rather limited, Weiss’s method fails to satisfy the timeliness. To solve this problem, this paper develops a DPD estimator using expectation maximization (EM) algorithm based on time delay and Doppler shift. The proposed method starts from choosing the transmitter-receiver range vector as the hidden variable. Then, the cost function is separated and simplified via the hidden variable, accomplishing the transformation from the high dimensional nonlinear search problem into a few one dimensional search subproblems. Finally, the expressions of EM repetition are obtained through Laplace approximation. In addition, we derive the Cramér–Rao bound to evaluate the best localization performance in this paper. Simulation results confirm that, on the basis of guaranteeing high accuracy, the proposed algorithm makes a good compromise in localization performance and computational complexity.

## 1. Introduction

The transmitter localization is a classic problem in wireless communication systems. It is well known that the conventional location approaches are composed of two separate steps: (1) The intermediate parameters are estimated through the signals. (2) The source position is determined from the measured parameters. In the past three decades, a great deal of work has been done in this field. Generally, the intermediate parameters (e.g., angle of arrival (AOA) [1], time of arrival (TOA) [2], time difference of arrival (TDOA) [3], Doppler shift [4,5,6] and frequency difference of arrival (FDOA) [7]) are usually estimated by the maximum likelihood (ML)-based method [8] or the subspace data fusion (SDF)-based method [5,9], and the position is mainly determined by an iterative algorithm [10] or a closed-form algorithm [11]. It is worth mentioning that Doppler information is a key parameter in the location problem based on the moving receiver. Gajewski [5] uses SDF criterion based on the Doppler effect to locate multiple emission sources. Since multipath propagation and Doppler effect are dominant factors to deteriorate localization performance, Kelner [6] uses the signal Doppler frequency method to resist non-line-of-sight (NLOS) conditions. As for the conventional approaches, it must be emphasized that the intermediate parameters are extracted by ignoring the constraint that all measurements must correspond to the same geolocation of the emitter, and more errors will be introduced in two separate processes of the conventional methods [12]. As a result, the conventional two-step methods cannot yield satisfactory location accuracy. For this reason, direct position determination (DPD) techniques that exploit the intrinsic constraint and determine the source position from the received signals directly were developed.

The DPD algorithms have been intensively investigated in recent years. An important classification of localization methods is related to propagation conditions, and there are two general cases: (1) multipath propagations with LOS and NLOS; (2) an additive white Gaussian noise (AWGN). In the first case, the methods [13,14,15,16] for LOS or NLOS environments have been deeply developed. Note that Du [16] proposed a DPD estimator for a novel localization architecture in multipath propagations, called the “Multiple Transponders and Multiple Receivers for Multiple Emitters Positioning System”. The second case is the most common condition, and the following discussion is also conducted under this assumption. Weiss [17] first proposed an SDF-based DPD algorithm with the utilization of orthogonality between signal subspace and noise subspace to estimate the emitter position. Chen [18] developed a multi-target DPD approach using subspace based on compressive sensing, reaching a high probability of locating the emitter without knowing the number of targets. Even if low computational complexity appears in the SDF-based DPD method, localization performance is deteriorated in low signal-to-noise ratios (SNRs), failing to reach the corresponding Cramér–Rao bound (CRB). To enhance the performance, ML-based DPD approaches were developed. Since the DPD cost function is often non-convex, traversal search is required to find the extremum. However, nuisance parameters (e.g., unknown transmission time [19] and timing errors [20]) will result in high dimensional search, which is impractical in a real-time application. To mitigate the computational load of traversal search, Weiss [21] proposed an ML-based approach via finding the maximum eigenvalue of the Hermitian matrix associated with the cost function, which also exhibits high localization accuracy. It must be emphasized that Weiss’s method is an ideal solution to account for localization accuracy and computation complexity in existing DPD algorithms. Moreover, the location performance can be further enhanced by utilizing signal properties. These DPD algorithms [22,23,24] can obtain superior localization precision by exploiting the constant modulus, orthogonal frequency division multiplexing, and the cyclostationary properties of signals. Unfortunately, the above DPD methods are not fast enough in the presence of limited computing resources, which is often a reality in moving receivers (i.e., airplanes or unmanned aerial vehicles (UAVs)). This limitation results in non-timeliness performance for moving emitter localization. On the other hand, DPD methods based on the ML criterion guarantee superior localization accuracy. Therefore, an ML-based DPD method with rapidity needs to be studied.

The expectation maximization (EM) algorithm is an attractive method of estimating the ML result when data can be divided into “incomplete data” and “complete data” in the model. In the past three decades, the EM algorithm has provided an excellent way to solve machine learning problems (i.e., speech processing and recognition [25] and image identification and restoration [26]). Via choosing the appropriate hidden variable, the EM algorithm decouples a high-dimensional search problem into a few subproblems with much lower computational complexity. In sight of the advantages of the EM algorithm, many scholars have applied it to the parameter estimation domain. Mada [27] uses the EM algorithm to solve the multi-source localization problem, leading to a soft computational load. Moreover, Lu estimates the source position via the EM algorithm for spatially nonwhite noise [28] and nonuniform noise [29], respectively, further demonstrating the effectiveness of the EM model in harsh scenarios. However, the DPD methods have not received the same treatment on the EM application, and only Tzoreff [30] discussed a DPD method based on the EM algorithm. Unfortunately, this method cannot suit for the moving-receiver scenario. As a result, there is a great demand for developing a DPD method using the EM algorithm for moving receivers.

This paper proposes a fast ML-based DPD algorithm using the EM algortihm based on time delay and Doppler shift, and the main processing steps are exhibited as follows:

(1) The transmitter-receiver range vector is selected as the hidden variable, successfully leading the separation and simplification of the ML cost function.
(2) With the help of Laplace approximation, the high-dimensional multi-parameter search of the prescribed ML estimator is decoupled into a closed form of the transmitter position and a line search of transmitter-receiver distance as well as transmitted time. Therefore, the expressions of EM repetition is determined.
(3) Iteration of the EM expressions, which alternately updates parameters in E-step and M-step, is required until the norm of the difference between the adjacent estimated position converges to a user’s predefined number.

In summary, the main contribution of this paper is the improvement of the prescribed ML DPD estimator via the EM algorithm in moving-receiver application for a far-field scenario, leading to a high effectiveness to find the global extreme. In addition, we also have derived the CRB to exhibit the best localization performance in theory. The rest of this paper is organized as follows. Section 2 lists the notations used in this paper. Section 3 constructs the signal model and formulates the problem. Section 4 discusses the DPD methods, including the previous method and the proposed method, and then makes a computational complexity comparison among different methods. Section 5 presents several numerical simulations to verify the theoretical analysis. Finally, Section 6 draws the conclusions.

## 2. Notations

In this section, some mathematical notation explanations that will be used through this paper are listed in Table 1.

## 3. Signal Model

This paper considers that *L* moving receivers intercepts the transmitted signal, and the signal is partitioned into *K* short intervals. Note that the antennas of the emitter and receivers are omnidirectional, and the positions of the emitter and receivers are determined in a two-dimensional coordinate system. In order to fully introduce the DPD model, three assumptions are included as follows:

**Assumption** **1.** *The receivers are moving. Let $p_{l,k}$ and $v_{l,k}$ denote the position and velocity of the lth receivers at the kth interception interval, which are precisely known to us. They are constant at each interception interval.*

**Assumption** **2.** *The stationary emitter, denoted by $p≜\left(x,y\right)$, locates in the far-field of the moving receivers.*

**Assumption** **3.** *The propagation channel is an AWGN channel, and time delay as well as Doppler information are used in the signal model.*

Based on the above assumptions, the complex signals $y_{l,k}\left(t\right)$ observed by the *l*th receiver at the *k*th interception interval at time *t* is expressed as [31]

(1) $$\begin{array}{c} y_{l,k}\left(t\right)=b_{l,k}s_{k}\left(t-\tau_{l,k}\left(p\right)-t_{0}\right)e^{j2\pi f_{l,k}t}+n_{l,k}\left(t\right)\;1\leq l\leq L;1\leq k\leq K \end{array}$$

where $t_{0}$ is the signal transmission time, and during the *k*th interception interval, $s_{k}\left(t\right)$ is the complex envelope of the emitter, $b_{l,k}$ denotes the channel attenuation, $n_{l,k}\left(t\right)$ is the white Gaussian, $\tau_{l,k}\left(p\right)$ is the propagation time between the emitter and the *l*th receiver, and Doppler frequency $f_{l,k}$ observed between the emitter and the *l*th receiver is given by [21]

(2) $$\begin{array}{c} f_{l,k}≜f_{c}+f_{c}\mu_{l,k}\left(p\right) \end{array}$$

where $f_{c}$ is the nominal frequency of the transmitted signal, assumed known, with

(3) $$\begin{array}{c} \mu_{l,k}\left(p\right)≜\frac{1}{c}\frac{v_{l,k}^{T}\left[p-p_{l,k}\right]}{∥p-p_{l,k}∥} \end{array}$$

where *c* is the radio wave propagation speed. It is assume that each receiver performs a down conversion of the intercepted signal by the nominal frequency. Thus, after down conversion the Doppler frequency can be replaced by

(4) $$\begin{array}{c} f_{l,k}≅f_{c}\mu_{l,k}\left(p\right). \end{array}$$

After sampling at $t=nT_{s}$, the received signal can be shown as

(5) $$\begin{array}{c} {\tilde{y}}_{l,k}\left(n\right)=b_{l,k}{\tilde{s}}_{k}\left(nT_{s}-\tau_{l,k}\left(p\right)-t_{k}\right)e^{j2\pi f_{l,k}nT_{s}}+{\tilde{n}}_{l,k}\left(n\right)\;n=1,2,\ldots ,N \end{array}$$

for l=1,⋯,L, and k=1,⋯,K, where *N* denotes the number of sample points at each interval. Then, Equation (5) can be expressed by a vector form as

(6) $$\begin{array}{c} {\tilde{y}}_{l,k}=b_{l,k}A_{l,k}{\tilde{s}}_{\tau_{l,k}}+{\tilde{n}}_{l,k} \end{array}$$

where

(7) $$\begin{array}{c} {\tilde{y}}_{l,k}≜{\left[{\tilde{y}}_{l,k}\left(1\right),{\tilde{y}}_{l,k}\left(2\right)\cdots ,{\tilde{y}}_{l,k}\left(N\right)\right]}^{T}\\ {\tilde{n}}_{l,k}≜{\left[{\tilde{n}}_{l,k}\left(1\right),{\tilde{n}}_{l,k}\left(2\right)\cdots ,{\tilde{n}}_{l,k}\left(N\right)\right]}^{T}\\ A_{l,k}=\mathrm{diag}\left\{\exp(-j2\pi f_{l,k}\tilde{N}T_{s})\right\}\\ {\tilde{s}}_{\tau_{l,k}}={\tilde{s}}_{k}{\left(\tilde{N}T_{s}-\tau_{l,k}\left(p\right)-t_{0}\right)}^{T}, \end{array}$$

with $\tilde{N}={\left[1,2,\ldots ,N\right]}^{T}$. We define ${\tilde{s}}_{k}={\left[{\tilde{s}}_{k}\left(T_{s}\right),{\tilde{s}}_{k}\left(2T_{s}\right),\cdots ,{\tilde{s}}_{k}\left(NT_{s}\right)\right]}^{T}$, and the Fourier coefficients of ${\tilde{s}}_{\tau_{l,k}}$ and ${\tilde{s}}_{k}$ satisfy

(8) $$\begin{array}{c} {\tilde{s}}_{\tau_{l,k}}=F^{H}D_{\tau_{l,k}}FF^{H}D_{t_{0}}F{\tilde{s}}_{k} \end{array}$$

where

(9) $$\begin{array}{c} F=\frac{1}{\sqrt{N}}\exp\left(-j\frac{2\pi }{N}\tilde{N}{\tilde{N}}^{T}\right)\\ D_{\tau_{l,k}}=\mathrm{diag}\left\{\exp\left(-j\frac{2\pi \tau_{l,k}}{NT_{s}}\tilde{N}\right)\right\}\\ D_{t_{0}}=\mathrm{diag}\left\{\exp\left(-j\frac{2\pi t_{0}}{NT_{s}}\tilde{N}\right)\right\}. \end{array}$$

F is the discrete Fourier transform operator. Therefore, Equation (6) is rewritten as

(10) $$\begin{array}{c} {\tilde{y}}_{l,k}=b_{l,k}C_{l,k}{\tilde{s}}_{k}+{\tilde{n}}_{l,k} \end{array}$$

where $C_{l,k}=A_{l,k}F^{H}D_{\tau_{l,k}}FF^{H}D_{t_{0}}F$.

## 4. Direct Position Determination Methods

This section discuss the DPD methods, which locate the emitter directly through the raw data. We first discuss the ML-based DPD method requiring traversal search, which can receive the best location accuracy. Weiss [19] proposed a fast ML-based DPD method based on eigenvalue decomposition. Unfortunately, the above methods cannot work effectively in the presence of limited computational sources. Thus, in light of the EM idea in estimation theory [27], a fast DPD method using EM algorithm will be proposed.

### 4.1. Previous Work

The DPD optimization based on time delay and Doppler shift is established, which was first introduced by [21]. The received signal ${\tilde{y}}_{l,k}$ is a complex Gaussian random vector. Hence, the likelihood function for $\tilde{y}$ can be formulated by [21]

(11) $$\begin{array}{c} l\left(\theta \right)=\frac{1}{{\left(\pi \sigma^{2}\right)}^{LKN}}\exp\left(-\frac{1}{\sigma^{2}}\sum_{k=1}^{K}\sum_{l=1}^{L}{∥{\tilde{y}}_{l,k}-b_{l,k}C_{l,k}{\tilde{s}}_{k}∥}^{2}\right) \end{array}$$

where $\sigma^{2}$ denotes the noise power, and $\theta ={\left[t_{0},b^{T},p^{T}\right]}^{T}$ denotes all unknown parameters, with ${b=\left[b_{1}^{T},b_{2}^{T},\cdots ,b_{K}^{T}\right]}^{T}$ and $b_{k}={\left[b_{1,k}^{T},b_{2,k}^{T},\cdots ,b_{L,k}^{T}\right]}^{T}$. The associated logarithmic likelihood function can be written as

(12) $$\begin{array}{c} L\left(\theta \right)=-LKN\ln\pi \sigma^{2}-\frac{1}{\sigma^{2}}\sum_{k=1}^{K}\sum_{l=1}^{L}{∥{\tilde{y}}_{l,k}-b_{l,k}C_{l,k}{\tilde{s}}_{k}∥}^{2}. \end{array}$$

Therefore, the estimation of noise power $\sigma^{2}$ is

(13) $$\begin{array}{c} \hat{\sigma^{2}}=\frac{1}{LKN}\sum_{k=1}^{K}\sum_{l=1}^{L}{∥{\tilde{y}}_{l,k}-b_{l,k}C_{l,k}{\tilde{s}}_{k}∥}^{2}. \end{array}$$

By substituting Equation (11) into Equation (10), the estimation of parameter θ can be determined by

(14) $$\begin{array}{c} \hat{\theta }=\underset{\theta }{\arg\min}\;f\left(\theta \right), \end{array}$$

with

(15) $$\begin{array}{c} f\left(\theta \right)=\sum_{k=1}^{K}\sum_{l=1}^{L}{∥{\tilde{y}}_{l,k}-b_{l,k}C_{l,k}{\tilde{s}}_{k}∥}^{2}. \end{array}$$

Since a high-dimensional nonlinear problem appears in Equation (14), it is difficult to compute the closed-form solution of $\hat{\theta }$. Thus, traversal searches are required among these stray parameters to obtain the best performance. However, this technique will take a long time to find the extreme corresponding to the emitter position, which is not efficient in practical application.

### 4.2. The Proposed Method

#### 4.2.1. EM Algorithm Review

The EM algorithm is an approach to iterative computation of the ML problem when the observations are regarded as incomplete data. In each iteration, it includes an expectation step and a maximization step. The meaning “incomplete data” reveals that there are two kinds of data: the incomplete data Y and the complete data X. More specifically, Y is the observed data, and X is the corresponding hidden data, (not observed directly). We denote the estimated parameter θ and an expression Y=f(X), which shows a many–one mapping from X to Y. Via the Bayesian rule, we have [30]

(16) $$\begin{array}{c} L\left(\theta \right)≜\log p_{Y}\left(Y;\theta \right)=\log p_{X,Y}\left(X,Y;\theta \right)-\log p_{X/Y}\left(X/Y;\theta \right) \end{array}$$

where $L\left(\theta \right)$ is the logarithmic likelihood function.

Since $p_{Y}\left(Y;\theta \right)$ is independent to X, the conditional expectation operation with associated with $p_{X/Y}\left(X/Y;\theta^{\prime }\right)$ will not make change to $L\left(\theta \right)$. The conditional expectation of Equation (14) can be expressed by

(17) $$\begin{array}{c} L\left(\theta \right)=E\left\{\log p_{X,Y}\left(X,Y;\theta \right)\right\}-E\left\{\log p_{X/Y}\left(X/Y;\theta \right)\right\} \end{array}$$

where $E\equiv E_{\left(X/Y;\theta^{\prime }\right)}\left\{•\right\}$ and $\theta^{\prime }$ denotes an arbitrary value of θ.

We define

(18) $$\begin{array}{c} Q\left(\theta ,\theta^{\prime }\right)=E\left\{\log p_{X,Y}\left(X,Y;\theta \right)\right\}\\ W\left(\theta ,\theta^{\prime }\right)=-E\left\{\log p_{X/Y}\left(X/Y;\theta \right)\right\}. \end{array}$$

Thus, Equation (17) can be rewritten as

(19) $$\begin{array}{c} L\left(\theta \right)=Q\left(\theta ,\theta^{\prime }\right)+W\left(\theta ,\theta^{\prime }\right). \end{array}$$

Based on the ML criterion, we can maximize $L\left(\theta \right)$ to estimate the unknown parameters. Since $p_{X/Y}\left(X/Y;\theta \right)$ is generally unknown to us, we need to approximate $W\left(\theta ,\theta^{\prime }\right)$.

Both sides of Equation (19) subtract $L\left(\theta^{\prime }\right)$ yielding

(20) $$\begin{array}{c} L\left(\theta \right)-L\left(\theta^{\prime }\right)=Q\left(\theta ,\theta^{\prime }\right)-Q\left(\theta^{\prime },\theta^{\prime }\right)+W\left(\theta ,\theta^{\prime }\right)-W\left(\theta^{\prime },\theta^{\prime }\right). \end{array}$$

With the help of Gibbs inequality, we find $W\left(\theta ,\theta^{\prime }\right)\geq W\left(\theta^{\prime },\theta^{\prime }\right)$. Thus, we obtain

(21) $$\begin{array}{c} L\left(\theta \right)-L\left(\theta^{\prime }\right)\geq Q\left(\theta ,\theta^{\prime }\right)-Q\left(\theta^{\prime },\theta^{\prime }\right). \end{array}$$

The above expression shows that an increment of *Q* associated with θ also ensures an increment of *L*. Therefore, the maximization problem of *L* is transformed into the maximization problem of *Q*. The EM algorithm contains two steps, and each iteration process can be given by

(22) $$\begin{array}{c} E-step:Calculate\;\;Q\left(\theta ,\theta^{\left(i\right)}\right),\\ M-step:\theta^{\left(i+1\right)}=\underset{\theta }{\arg\max}\;Q\left(\theta ,\theta^{\left(i\right)}\right). \end{array}$$

An iteration cycle exists in Equation (20). We obtain $\theta^{\left(i+1\right)}$ in a current iteration step, and it will be the initial value to repeat the EM operation of Equation (20) in the next iteration step. When *Q* converges, the iteration stops.

#### 4.2.2. Derivation of the EM-DPD Algorithm

After the above analysis, we choose the received signals $\tilde{Y}={\left[{\tilde{y}}_{1,1}^{T},\cdots ,{\tilde{y}}_{l,k}^{T},\cdots ,{\tilde{y}}_{L,K}^{T}\right]}^{T}$ as the observed data and the transmitter-receiver range vectors $\tilde{X}={\left[{\tilde{x}}_{1,1}^{T},\cdots ,{\tilde{x}}_{l,k}^{T},\cdots ,{\tilde{x}}_{L,K}^{T}\right]}^{T}$ as the hidden data, respectively. We assume that ${\tilde{x}}_{l,k}$ is a Gaussian vector, and the probability distribution function (PDF) of $\tilde{X}$ can be shown as

(23) $$\begin{array}{c} p_{\tilde{X}}\left(\tilde{X};p\right)=\prod_{k=1}^{K}\prod_{l=1}^{L}p_{\tilde{x}}\left({\tilde{x}}_{l,k};p\right)=\prod_{k=1}^{K}\prod_{l=1}^{L}N\left(\varepsilon_{l,k}\left(p\right),\sigma_{x}^{2}I_{2}\right) \end{array}$$

where $\varepsilon_{l,k}\left(p\right)=p-p_{l,k}$ and $\sigma_{x}^{2}$ is the variance of *x*. We define $G_{l,k}≜C_{l,k}{\tilde{s}}_{k}$, and Equation (6) is rewritten as

(24) $$\begin{array}{c} {\tilde{y}}_{l,k}=b_{l,k}G_{l,k}+{\tilde{n}}_{l,k}. \end{array}$$

We assume that ${\tilde{n}}_{l,k}$ are complex Gaussian vectors with mean $0_{N}$ and covariance $\sigma^{2}I_{N}$. Therefore, the pdf of ${\tilde{y}}_{l,k}$ is given by

(25) $$\begin{array}{c} p_{\tilde{y}}\left({\tilde{y}}_{l,k};p,t_{0}\right)=\frac{1}{{\left(\pi \sigma^{2}\right)}^{N}}\exp\left\{-\frac{{∥{\tilde{y}}_{l,k}-b_{l,k}G_{l,k}∥}^{2}}{\sigma^{2}}\right\}. \end{array}$$

Assuming the signals observed by different receivers and different observations intervals are independent, we have

(26) $$\begin{array}{c} p_{\tilde{Y}}\left(\tilde{Y};p,t_{0}\right)=\prod_{k=1}^{K}\prod_{l=1}^{L}p_{\tilde{y}}\left({\tilde{y}}_{l,k};p,t_{0}\right)\propto \prod_{k=1}^{K}\prod_{l=1}^{L}\exp\left\{-\sigma^{2}{∥{\tilde{y}}_{l,k}-b_{l,k}G_{l,k}∥}^{2}\right\}. \end{array}$$

Note that the source coordinates p is the direct relation parameter to the observations $\tilde{Y}$. We need to separate this variable to the subsequent derivation. By introducing $\tilde{X}$ in Equation (23) and defining $\phi ={\left[t_{0},b^{T}\right]}^{T}$, Equation (26) can be rewritten as

(27) $$\begin{array}{c} p_{\tilde{Y}}\left(\tilde{Y}/\tilde{X};\phi \right)=\prod_{k=1}^{K}\prod_{l=1}^{L}p_{\tilde{y}/\tilde{x}}\left({\tilde{y}}_{l,k}/{\tilde{x}}_{l,k},b_{l,k},t_{0}\right)\propto \prod_{k=1}^{K}\prod_{l=1}^{L}\exp\left\{-\sigma^{2}{∥{\tilde{y}}_{l,k}-b_{l,k}G_{l,k}∥}^{2}\right\} \end{array}$$

where $G_{l,k}\equiv G_{l,k}\left({\tilde{x}}_{l,k},t_{0}\right)$ for brevity.

From the analysis in Section 4.2.1, we can estimate the emitter position via maximizing the auxiliary function $Q\left(\theta ,\theta^{\prime }\right)$. Next we introduce the key procedure of the derivation.

**Proposition** **1.** *The auxiliary function $Q\left(\theta ,\theta^{\prime }\right)$ is separated into*

(28) $$\begin{array}{c} Q\left(\theta ,\theta^{\prime }\right)=Q_{1}\left(\phi ,\phi^{\prime }\right)+Q_{2}\left(p,p^{\prime }\right). \end{array}$$

**Proof of Proposition.**  Via the Bayesian rule, the joint probability $p_{\tilde{X},\tilde{Y}}\left(\tilde{X},\tilde{Y};\theta \right)$ is expressed by the product of Equations (23) and (26), which depend only on p and only on ϕ, respectively. Since the log(•) of $p_{\tilde{X},\tilde{Y}}\left(\tilde{X},\tilde{Y};\theta \right)$ exists in Equation (18), the separation of $Q\left(\theta ,\theta^{\prime }\right)$ can be shown as

(29) $$\begin{array}{c} Q_{1}\left(\phi ,\phi^{\prime }\right)=-\sigma^{-2}\sum_{k=1}^{K}\sum_{l=1}^{L}E\left\{{∥{\tilde{y}}_{l,k}-b_{l,k}G_{l,k}∥}^{2}\right\} \end{array}$$

(30) $$\begin{array}{c} Q_{2}\left(p,p^{\prime }\right)=-\sigma_{x}^{-2}\sum_{k=1}^{K}\sum_{l=1}^{L}E\left\{{∥{\tilde{x}}_{l,k}-\varepsilon_{l,k}\left(p\right)∥}^{2}\right\}. \end{array}$$

By maximizing Equation (29), we obtain

(31) $$\begin{array}{c} {\hat{b}}_{l,k}=\frac{{\bar{G}}_{l,k}^{H}{\tilde{y}}_{l,k}}{E_{s}}\;\forall \;l=1,\cdots ,L;\;k=1,\cdots ,K \end{array}$$

where ${\bar{G}}_{l,k}\equiv E\left\{G_{l,k}\right\}$ and $E_{s}\equiv {∥G_{l,k}∥}^{2}$. Substituting Equation (31) into Equation (29) yields

(32) $$\begin{array}{c} Q_{1}\left(t_{0},t_{0}^{\prime }\right)=-\sigma^{-2}\sum_{k=1}^{K}\sum_{l=1}^{L}{\tilde{y}}_{l,k}^{H}\Psi_{l,k}{\tilde{y}}_{l,k} \end{array}$$

where $\Psi_{l,k}=I_{N}-E_{s}^{-1}{\bar{G}}_{l,k}{\bar{G}}_{l,k}^{H}$. We continue to maximize the above expression, yielding

(33) $$\begin{array}{c} {\hat{t}}_{0}=\underset{t_{0}}{\arg\max}\;\sum_{k=1}^{K}\sum_{l=1}^{L}{\left|{\tilde{y}}_{l,k}^{H}{\bar{G}}_{l,k}\left(t_{0}\right)\right|}^{2}. \end{array}$$

Similarly, maximizing Equation (28) with respect to p yields

(34) $$\begin{array}{cc} \hat{p} & =\underset{p}{\arg\min}\;\sum_{k=1}^{K}\sum_{l=1}^{L}{∥p_{l,k}-p∥}^{2}-2p^{T}{\bar{x}}_{l,k}\\ & =\frac{1}{KL}\sum_{k=1}^{K}\sum_{l=1}^{L}\left(p_{l,k}-{\bar{x}}_{l,k}\right). \end{array}$$

After eliminating b in the optimization process, we obtain the ML estimated expression of $t_{0}$ and the closed-form solution of p through the EM procedure. Note that ${\bar{G}}_{l,k}$ in Equation (31) and ${\bar{x}}_{l,k}$ in Equation (32) are need to estimate. This work is promoted by Laplace approximation, which is shown in Appendix A. We define $r_{l,k}≜∥x_{l,k}∥$, $R_{l,k}≜∥\varepsilon_{l,k}∥$, and $\lambda =\frac{\sigma_{x}^{2}}{\sigma^{2}}$, the expression of the EM-DPD algorithm is written as follows:

E-step:

(35) $$\begin{array}{c} \;r_{l,k}^{*}=\underset{r_{l,k}}{\arg\max}\;-\frac{r_{l,k}^{2}}{2}+R_{l,k}r_{l,k}-\lambda {∥{\tilde{y}}_{l,k}-{\hat{b}}_{l,k}G_{l,k}\left(r_{l,k}\right)∥}^{2}\\ \;\forall l=1,\cdots ,L;\;k=1,\cdots ,K. \end{array}$$

M-step:

(36) $$\begin{array}{c} \;p^{\left(i+1\right)}=\frac{1}{KL}\sum_{k=1}^{K}\sum_{l=1}^{L}\left[p_{l,k}\left(1-\frac{r_{l,k}^{*}}{R_{l,k}}\right)+\frac{r_{l,k}^{*}}{R_{l,k}}p^{\left(i\right)}\right]\;. \end{array}$$

(37) $$\begin{array}{c} \;t_{0}^{\left(i+1\right)}=\underset{t_{0}}{\arg\max}\;\sum_{k=1}^{K}\sum_{l=1}^{L}{\left|{\tilde{y}}_{l,k}^{H}{\bar{G}}_{l,k}\left(r_{l,k}^{*},t_{0}^{\left(i\right)}\right)\right|}^{2}\;. \end{array}$$

□

For easy understanding, the main steps of the proposed method are exhibited in Algorithm 1.

**Algorithm 1:** The main steps of the proposed method.
**Input**:
The observed data: ${\tilde{y}}_{l,k}$, the position, and the velocity of receiver: $p_{l,k}$ and $v_{l,k}$, (l=1,⋯,L,k=1,⋯,K);
1. Choose a small positive number ε>0, and set the iteration counter to i=1;
2. Set i = 0, initialize $p^{\left(i\right)}$, $t_{0}^{\left(i\right)}$;
3. Calculate $r_{l,k}^{*}\left(l=1,\cdots ,L,k=1,\cdots ,K\right)$ via Equation (35) in E-step;
4. Substitute $r_{l,k}^{*}\left(l=1,\cdots ,L,k=1,\cdots ,K\right)$ into Equations (36) and (37) to obtain $p^{\left(i+1\right)}$ and $t_{0}^{\left(i+1\right)}$ through M-step, respectively. And then set i=i+1;
5. Calculate Δ=$∥p^{\left(i\right)}-p^{\left(i-1\right)}∥$. If Δ≤ε, stop the iterations; Otherwise, set i=i+1, repeat steps 3–4;
**Output**: The estimated position of target $\hat{p}=p^{\left(i\right)}$.

**Remark** **1.** *By choosing the hidden data X, the high-dimensional nonlinear problem in Equation (14) is simplified as a few subproblems with soft computational load. The final operations are the line search of $r_{l,k}$ in the E-step and the closed-form solution of p and the line search of $t_{0}$ in the M-step.*

### 4.3. Computational Complexity Analysis

Based on the above analysis, we have achieved the transformation from high-dimensional multi-parameter nonlinear problem into several optimization subproblems, whose computational complexity is substantially reduced. Nonetheless, the computational complexity differs among the traversal search method, Weiss’s method [21], and the proposed method. The traversal search method requires a three-dimensional search to find the extreme of the cost function corresponding to the emitter position. Weiss’s method mainly has a two-dimensional search of the maximum eigenvalue of a Hermitian matrix. The proposed method is dominated by the line searches of $r_{l,k}^{*}\left(l=1,\cdots ,L,k=1,\cdots ,K\right)$ in Equation (35) and $t_{0}$ in Equation (37).

To better exhibit the computational complexity, Table 2 lists the computational complexity of the traversal search method, Weiss’s method, and the proposed method. Note that $N_{p}$ is the number of grid search points with respect to a line search, and $I_{iter}$ is the number of iterations of the proposed method. It must be emphasized that the key contributor to computational complexity is $N_{p}$. Compared with the two- or three-dimensional searches in other methods, the proposed method only has a one-dimensional search, which reduces the computational complexity significantly.

## 5. Numerical Experiments

In this section, several numerical experiments are reported to corroborate the theoretical analysis. All simulations results are based on 200 Monte Carlo trials. In this scenario, the receivers equipped with only one sensor are included, and the source emits a Gaussian random signal with a center frequency of $f_{c}$ = 200 MHz. The channel attenuation coefficient amplitude obeys a normal distribution with a mean of 1 and a standard deviation of 0.1, and the channel phase obeys a uniform distribution over [−π,π]. Unless otherwise specified, we collect N=32 sample points in each interval at a sampling rate of $f_{s}$ = 400 kHz, use *L* = 3 receivers, perform a total of *K* = 5 observations, and set the velocity of receiver *v* = 300 m/s.

To examine the performance comparisons, we take simulations works with the following four algorithms

1. the proposed method in this study;
2. the traversal search method;
3. Weiss’s method;
4. the TOA/Doppler two-step algorithm.

The details of the comparison algorithms are shown in Table 3.

Additionally, the CRB presented in Appendix B is also included in this part, providing a theoretical best performance benchmark. Moreover, root mean square error (RMSE) and cumulative distribution function (CDF) are adopted to evaluate localization accuracy in this paper.

**Figure 1:**
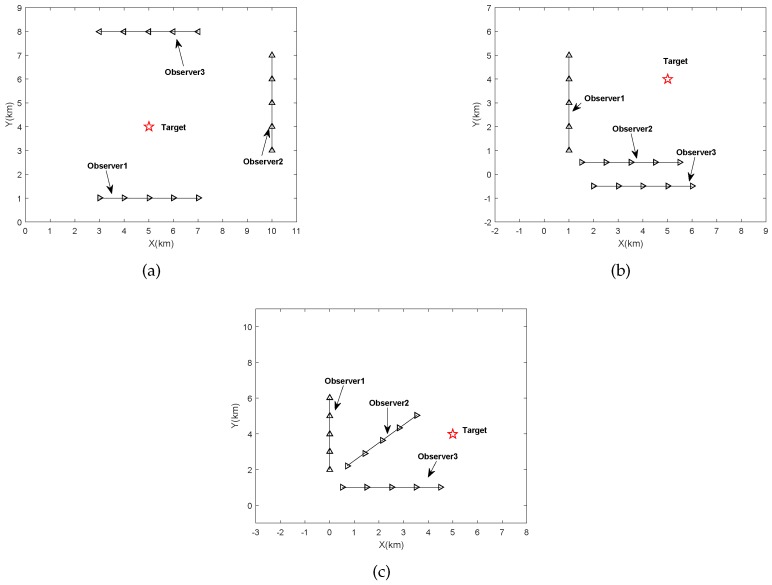
The simulated localization scenarios: (**a**) scenario 1, (**b**) scenario 2, (**c**) scenario 3.

**Figure 2:**
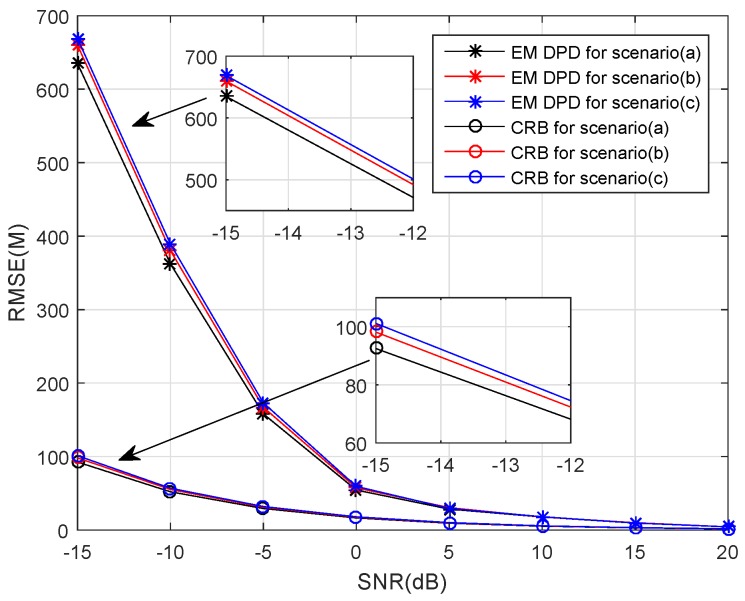
The RMSE and Cramér–Rao bound (CRB) versus SNRs for different scenarios.

We now examine the localization performance of the proposed algorithm for three different scenarios. The target locates at [5, 4] km, and the receivers move along different trajectories, which can be found in Figure 1. As shown in Figure 2, the performance of our algorithm is not sensitive to the receiver trajectories because there is no significant difference between the RMSE curves of our algorithm for different scenarios. Additionally, as expected, the proposed method for Scenario (a) has better localization precision in low SNRs. On the other hand, although the RMSE of our algorithm is far from that of the corresponding CRB in low SNRs, it has a substantial advantage in computation time (see the descriptions for Table 4).

**Figure 3:**
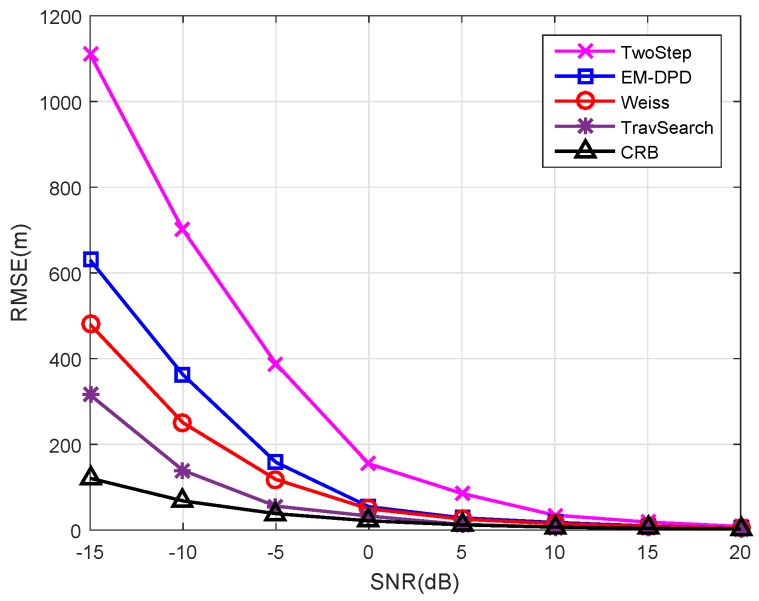
The RMSEs of different algorithms versus SNRs.

It must be emphasized that the next simulations are all based on Scenario (a) in Figure 1. Firstly, we continue to conduct the simulation for algorithm performance comparison versus SNRs. The RMSEs of all algorithms can be easily found in Figure 3. Unsurprisingly, all DPD methods outperform the two-step method, and the traversal search method shows the best localization performance especially in low SNRs. Although the performance of the proposed method is slightly inferior than that of Weiss’s method in low SNRs, it is more efficient. However, the other two DPD methods will cost a high amount of time in the presence of limited computing resources, losing its timeliness to moving target. The detailed runtime information can be seen in Table 4.

Secondly, to better exhibit the computational complexity of each algorithm, the runtime is investigated. From the results in Table 4, we observe that the two-step method requires the least runtime, but its localization performance is deteriorated in low SNRs (see Figure 3). Due to the high-dimensional search, the traversal search method is more computationally expensive than other DPD methods. Obviously, Weiss’s method has a great improvement in the running time. Compared with Weiss’s method, the run time of our method is further reduced (approximately 40%). However, the reduction of computational complexity of our method does not deteriorate localization performance significantly (also see Figure 3). As indicated by these finding, the proposed approach receives acceptable localization performance with a low computation resource cost.

**Figure 4:**
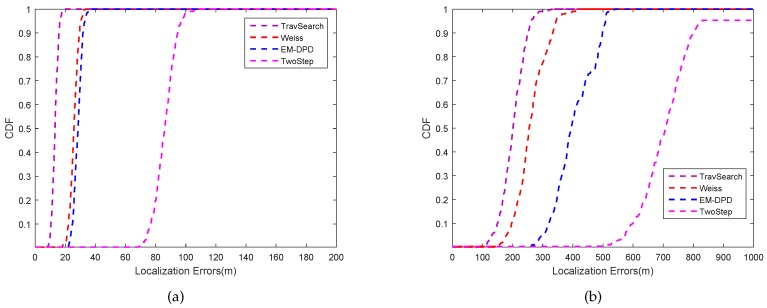
The CDF of different algorithms versus localization errors: (**a**) SNR = 5 dB; (**b**) SNR = −10 dB.

Thirdly, the CDF curves of all methods at the nominal constellation are depicted in Figure 4a,b at the SNR level of 5 dB and −10 dB, respectively. It can be readily observed that the traversal search curve is highest at a different localization error level. The proposed method curve is associated with Weiss’s DPD curve, which indicates it can receive high localization accuracy with high SNRs. From Figure 4, the two-step method curve deviates from the DPD methods. The proposed method curve is slightly apart from the curve of Weiss’s method, which is acceptable in terms of low complexity.

**Figure 5:**
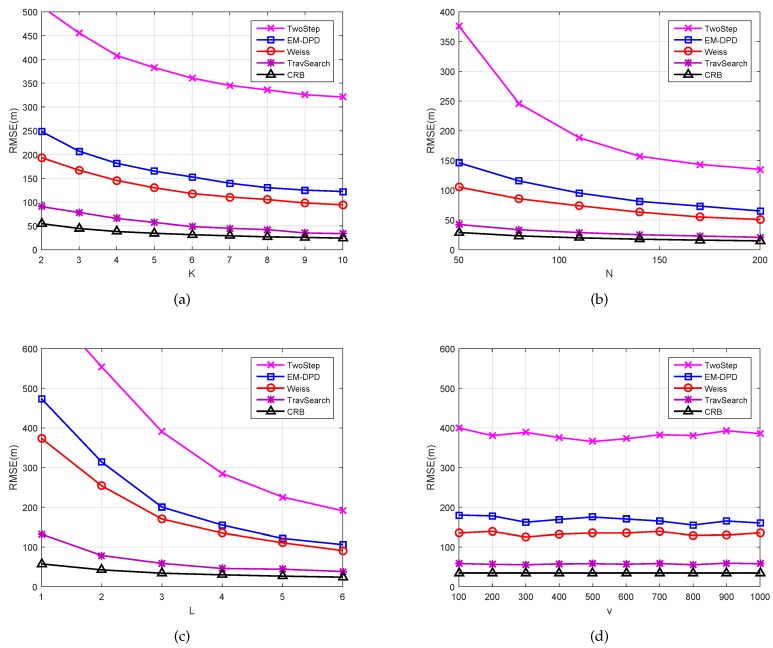
The RMSEs of different algorithms versus system parameters: (**a**) the value of *K*; (**b**) the value of *N*; (**c**) the value of *L*; (**d**) the value of *v*.

Finally, to further examine the influence of system parameter values on localization performance, we set SNR = −5 dB and exhibit the RMSE curves for *K*, *N*, *L*, and *v* in Figure 5, respectively. Additionally, the simulation conditions are *K* ranging from 2 to 10, *N* ranging from 50 to 200, *L* ranging from 1 to 6, and *v* ranging from 100 to 1000. It can be readily found in Figure 5a–c that all algorithms perform better in terms of localization accuracy, as more system parameter information exists. These results imply that the increase of these system parameters can enhance the localization performance. However, more parameter requirements mean greater system overhead and computational pressure. Consequently, our method can play an excellent role in the presence of limited computational resources. Furthermore, Figure 5d indicates that *v* does not have much impact on position accuracy.

## 6. Conclusions

The traditional two-step methods have high computational efficiency but low localization performance. To enhance positioning accuracy, DPD approaches have been developed. Since the DPD technology locates the target from the signal directly, computational complexity of this estimator is high. To solve this problem, this paper proposes a fast ML-based direct localization method using an EM algorithm based on time delay and Doppler shift. The EM scheme was developed to solve the ML problem in the DPD model. We simplify this ML problem via the theories of Gibbs inequality and Laplace approximation, transforming the high-dimensional nonlinear search into a few one-dimensional searches. Simulation results show that the proposed algorithm has operates faster than other DPD methods. Furthermore, when limited computing resources appear, our method leads a more efficient result on the basis of guaranteeing high localization accuracy. Therefore, compared with other localization approaches, the proposed method becomes a good way to balance localization accuracy and computational complexity.

## Figures and Tables

**Table 1:** Mathematical notation explanation.

Notation	Explanation
${\left[•\right]}^{T}$	transpose
${\left[•\right]}^{H}$	conjugate transpose
${•}^{\left(R\right)}$	the real part
${•}^{\left(I\right)}$	the imaginary part
diag{•}	a diagonal matrix with diagonal entries
⊗	Kronecker product
∥•∥	Euclidean norm of the matrix
$\left|•\right|$	determinant of the matrix
$p_{X,Y}\left\{•\right\}$	the joint distribution of X and Y
$p_{X/Y}\left\{•\right\}$	the conditional distribution of X given Y
$I_{N}$	N×N identity matrix
$0_{N}$	N×N matrix with zero

**Table 2:** Computational complexity.

Algorithm	Amount of Computation
Traversal search method	$O\left(N_{p}^{3}KL\left(8N^{3}+7N^{2}+N\right)\right)$
Weiss’s method	$O\left(N_{p}^{2}\left(K\left(\left(5L+3\right)N^{3}+3N^{2}+2L^{3}\right)\right)\right)$
Proposed method	$O\left(I_{iter}\left(N_{p}KL\left(14N^{3}+8N^{2}+3N\right)+KL\right)\right)$

**Table 3:** Algorithms.

Algorithm	Method
TOA/Doppler two-step method	Method in [2] to estimate TOA; Method in [8] to estimate Doppler; Least square (LS) location using the TOA and Doppler estimations.
Traversal search method	Method in [17] using a three-dimensional search
Weiss’s method	Method in [21] using eigenvalue decomposition

**Table 4:** Mean runtime of different methods.

Algorithm	Runtime (s)
Two-step method	0.8
Traversal search method	67
Weiss’s method	7.9
Proposed method	4.7

## Appendix A. Evaluation of x*_l,k_* and ${\bar{G}}_{l,k}$ via Laplace Method

We consider the multivariate functions $h\left(z\right):\;R^{n}\to R^{m}$ and $f\left(z\right):\;R^{n}\to R$. An integral is expressed by

(A1) $$\begin{array}{c} I_{f}=\int_{z\in Z}h\left(z\right)e^{f\left(z\right)}dz. \end{array}$$

Assume that $f\left(z\right)$ has a global maximum within the interval. According to [27], the Laplace approximation is written as

(A2) $$\begin{array}{c} I_{f}\approx {\left(2\pi \right)}^{n/2}{\left|-F\left(z^{*}\right)\right|}^{-1/2}h\left(z^{*}\right)e^{f\left(z^{*}\right)} \end{array}$$

where $F\left(z^{*}\right)$ is the Hessian of $f\left(z\right)$ at $z^{*}$. Next we approximate the integral of ${\bar{x}}_{l,k}$ and ${\bar{G}}_{l,k}$. For the sake of brevity, we omit l,k in the subsequent derivation.

The expression of the conditional expectation is

(A3) $$\begin{array}{c} \bar{h}\left(\tilde{x}\right)≜\int h\left(\tilde{x}\right)p_{\tilde{x}/\tilde{y}}d\tilde{x}=\int h\left(\tilde{x}\right)\frac{p_{\tilde{x},\tilde{y}}}{p_{\tilde{y}}}d\tilde{x}\\ \propto p_{\tilde{y}}^{-1}\int h\left(\tilde{x}\right)e^{-\frac{{∥\tilde{x}-\varepsilon \left(p\right)∥}^{2}}{\sigma_{x}^{2}}-\frac{{∥\tilde{y}-\hat{b}G\left(\tilde{x}\right)∥}^{2}}{\sigma^{2}}}d\tilde{x}. \end{array}$$

The polar coordinates can be written as

(A4) $$\begin{array}{c} \tilde{x}=r\left[\begin{array}{c} \cos\xi \\ \sin\xi \end{array}\right],\;\varepsilon =R\left[\begin{array}{c} \cos\eta \\ \sin\eta \end{array}\right]. \end{array}$$

Substituting (A4) into (A3) yields

(A5) $$\begin{array}{c} \bar{h}\left(r,\xi \right)\propto p_{\tilde{y}}^{-1}\int_{0}^{\infty }re^{f\left(r\right)}dr\int_{-\pi }^{\pi }h\left(r,\xi \right)e^{\alpha r\cos\left(\xi -\eta \right)}d\xi \end{array}$$

where $f\left(r\right)=-\sigma_{x}^{-2}\left(\frac{r^{2}}{2}-\lambda {∥\tilde{y}-\hat{b}G\left(r\right)∥}^{2}\right)$, $\lambda =\frac{\sigma_{x}^{2}}{\sigma^{2}}$ and $\alpha =R\sigma_{x}^{-2}$. We assume r≫1 and have [28]

(A6) $$\begin{array}{c} I_{1}\left(r\right)=\frac{1}{2\pi }\int_{-\pi }^{\pi }\cos\xi e^{r\cos\xi }d\xi \approx \frac{e^{r}}{\sqrt{2\pi r}},\\ I_{0}\left(r\right)=\frac{1}{2\pi }\int_{-\pi }^{\pi }e^{r\cos\xi }d\xi \approx \frac{e^{r}}{\sqrt{2\pi r}},\\ 0=\int_{-\pi }^{\pi }\sin\xi e^{r\cos\xi }d\xi \end{array}$$

where $I_{1}\left(•\right)$, $I_{0}\left(•\right)$ denote the first kind of modified Bessel function. We substitute Equation (A6) into Equation (A5), yielding

(A7) $$\begin{array}{c} \bar{\tilde{x}}\propto \left[\begin{array}{c} \cos\eta \\ \sin\eta \end{array}\right]\int_{0}^{\infty }r^{3/2}e^{f\left(r\right)+\alpha r}dr. \end{array}$$

Recalling the expression in Equation (A2), we can search for the global extreme $r^{*}$ through

(A8) $$\begin{array}{c} r^{*}=\underset{r}{\arg\max}\;f\left(r\right)+\alpha r. \end{array}$$

Therefore, we have

(A9) $$\begin{array}{c} \bar{\tilde{x}}\approx \left[\begin{array}{c} \cos\eta \\ \sin\eta \end{array}\right]r^{*}=\frac{\varepsilon }{∥\varepsilon ∥}r^{*}. \end{array}$$

Similarly, we have $\bar{G}\approx G\left(r^{*}\right)$.

## Appendix B. Derivation of the Cramér–Rao Bound

It is well known that the CRB provides a benchmark for the best localization accuracy for any unbiased estimator. This section derives the compact CRB formula for source position with known signal waveforms and unknown transmitted time.

Let $D_{l,k}\left(\theta \right)=b_{l,k}C_{l,k}{\tilde{s}}_{k}$. According to [22], the q,jth entry of the fisher information matrix of unknown parameter θ is shown as

(A10) $$\begin{array}{c} {\left[J\right]}_{q,j}=\frac{2}{\sigma^{2}}\sum_{k=1}^{K}\sum_{l=1}^{L}{\left(\frac{\partial D_{l,k}\left(\theta \right)}{\partial {\left[\theta \right]}_{q}^{T}}\right)}^{H}\left(\frac{\partial D_{l,k}\left(\theta \right)}{\partial {\left[\theta \right]}_{j}^{T}}\right). \end{array}$$

For easy derivation, θ can be redefined by

(A11) $$\begin{array}{c} \theta ={\left[b^{\left(R\right)T},b^{\left(I\right)T},t_{0},p^{T}\right]}^{T} \end{array}$$

where ${b=\left[b_{1}^{T},b_{2}^{T},\cdots ,b_{K}^{T}\right]}^{T}$ and $b_{k}={\left[b_{1,k}^{T},b_{2,k}^{T},\cdots ,b_{L,k}^{T}\right]}^{T}$. Thus, J can be expressed by

(A12) $$\begin{array}{c} J=\left[\begin{array}{cccc} J_{\mathrm{pp}} & J_{pt_{0}} & J_{pb^{\left(R\right)}} & J_{pb^{\left(I\right)}}\\ J_{t_{0}p} & J_{t_{0}t_{0}} & J_{t_{0}b^{\left(R\right)}} & J_{t_{0}b^{\left(I\right)}}\\ J_{b^{\left(R\right)}p} & J_{b^{\left(R\right)}t_{0}} & J_{b^{\left(R\right)}b^{\left(R\right)}} & J_{b^{\left(R\right)}b^{\left(I\right)}}\\ J_{b^{\left(I\right)}p} & J_{b^{\left(I\right)}t_{0}} & J_{b^{\left(I\right)}b^{\left(R\right)}} & J_{b^{\left(I\right)}b^{\left(I\right)}} \end{array}\right]. \end{array}$$

Next we calculate the subblocks of Equation (A12).

### Appendix B.1. The Partial of D_l,k_ (θ) with Respect to b

Note that b is a complex variable, so we obtain

(A13) $$\begin{array}{c} {\left[\frac{\partial D_{l,k}\left(\theta \right)}{\partial b^{\left(R\right)T}}\right]}_{m,n}=\left\{\begin{array}{cc} G_{l,k} & m=l,n=k\\ 0 & m\neq l,n\neq k \end{array}\right.,\\ {\left[\frac{\partial D_{l,k}\left(\theta \right)}{\partial b^{\left(I\right)T}}\right]}_{m,n}=\left\{\begin{array}{cc} jG_{l,k} & m=l,n=k\\ 0 & m\neq l,n\neq k \end{array}\right.. \end{array}$$

### Appendix B.2. The Partial of D_l,k_ (θ) with Respect to t_0_

We obtain

(A14) $$\begin{array}{c} \frac{\partial D_{l,k}\left(\theta \right)}{\partial t_{0}}=H_{l,k}, \end{array}$$

where $H_{l,k}=b_{l,k}A_{l,k}F^{H}D_{\tau_{l,k}}FF^{H}{\overset{•}{D}\;}_{t_{0}}F{\tilde{s}}_{k}$, with ${\overset{•}{D}\;}_{t_{0}}=D_{t_{0}}\cdot \mathrm{diag}\left\{-j2\pi \frac{\tilde{N}}{NT_{s}}\right\}$.

### Appendix B.3. The Partial of D_l,k_ (θ) with Respect to p

We define $\Gamma_{l,k}=\frac{\partial A_{l,k}F^{H}D_{T_{l,k}}F}{\partial p^{T}}\left(I_{2}⊗{\tilde{s}}_{k}^{t_{0}}b_{l,k}\right)$ with ${\tilde{s}}_{k}^{t_{0}}={\left[{\tilde{s}}_{k}\left(T_{s}-t_{0}\right),{\tilde{s}}_{k}\left(2T_{s}-t_{0}\right),\cdots ,{\tilde{s}}_{k}\left(NT_{s}-t_{0}\right)\right]}^{T}$. The derivative with respect to p can be expressed by

(A15) $$\begin{array}{c} \frac{\partial D_{l,k}\left(\theta \right)}{\partial p^{T}}=\Gamma_{l,k} \end{array}$$

where

(A16) $$\begin{array}{c} \frac{\partial A_{l,k}F^{H}D_{T_{l,k}}F}{\partial p^{T}}=\frac{\partial A_{l,k}}{\partial p^{T}}\left(I_{2}⊗F^{H}D_{\tau_{l,k}}F\right)+A_{l,k}F^{H}\frac{\partial D_{\tau_{l,k}}}{\partial p^{T}}\left(I_{2}⊗F\right), \end{array}$$

with

(A17) $$\begin{array}{c} \frac{\partial A_{l,k}}{\partial p^{T}}=A_{l,k}\cdot \mathrm{diag}\left\{j2\pi \frac{\partial f_{l,k}}{\partial p^{T}}T_{s},j2\pi \frac{\partial f_{l,k}}{\partial p^{T}}2T_{s},\ldots ,j2\pi \frac{\partial f_{l,k}}{\partial p^{T}}NT_{s}\right\} \end{array}$$

(A18) $$\begin{array}{c} \frac{\partial f_{l,k}}{\partial p^{T}}=\frac{f_{c}}{c}\left(\frac{v_{l,k}^{T}}{∥p-p_{l,k}∥}-\frac{v_{l,k}^{T}\left(p-p_{l,k}\right){\left(p-p_{l,k}\right)}^{T}}{{∥p-p_{,k}∥}^{3}}\right) \end{array}$$

(A19) $$\begin{array}{c} \frac{\partial D_{\tau_{l,k}}}{\partial p^{T}}=D_{\tau_{l,k}}\cdot \mathrm{diag}\left\{-j2\pi \frac{1}{NT_{s}}\frac{\partial \tau_{l,k}}{\partial p^{T}},-j2\pi \frac{2}{NT_{s}}{\frac{\partial \tau_{l,k}}{\partial p^{T}}}_{s},\ldots ,-j2\pi \frac{N}{NT_{s}}{\frac{\partial \tau_{l,k}}{\partial p^{T}}}_{s}\right\} \end{array}$$

(A20) $$\begin{array}{c} \frac{\partial \tau_{l,k}}{\partial p^{T}}=\frac{1}{c}\frac{{\left(p-p_{l,k}\right)}^{T}}{∥p-p_{l,k}∥}. \end{array}$$

By substituting Equations (A13)–(A15) into Equation (A10), we can obtain the subblocks of $J_{\xi \xi }$

(A21) Jbm1,n1(R)bm2,n2(R)=2σ2ReGm1,n1HGm2,n2m1=m2∩n1=n20m1≠m2∪n1≠n2.

(A22) Jbm1,n1(R)bm2,n2(I)=−2σ2ImGm1,n1HGm2,n2m1=m2∩n1=n20m1≠m2∪n1≠n2.

(A23) Jbm,nRt0=2σ2ReGm,nHHm,n.

(A24) Jbm,nRp=2σ2ReGm,nHΓm,n.

(A25) Jbm1,n1(I)bm2,n2(I)=2σ2ReGm1,n1HGm2,n2m1=m2∩n1=n20m1≠m2∪n1≠n2.

(A26) Jbm,nIt0=−2σ2ImGm,nHHm,n.

(A27) Jbm,nIp=−2σ2ImGm,nHΓm,n.

(A28) Jt0t0=2σ2∑l=1L∑k=1KReHl,kHHl,k.

(A29) Jt0p=2σ2∑k=1K∑l=1LReHl,kHΓl,k.

(A30) Jpp=2σ2∑l=1L∑k=1KReΓl,kHΓl,k.

Since submatrices of Equation (A12) with respect to diagonal symmetry are transposed matrices of each other, we can easily obtain the remaining submatrices.

Thus, the inverse of the CRB of position can be obtained by

(A31) $$\begin{array}{cc} {\left(\mathrm{CR}B_{p}\right)}^{-1}=J_{\mathrm{pp}}-\left[\begin{array}{ccc} J_{pb^{\left(R\right)}} & J_{pb^{\left(I\right)}} & J_{pt_{0}} \end{array}\right] & {\left[\begin{array}{ccc} J_{b^{\left(R\right)}b^{\left(R\right)}} & J_{b^{\left(R\right)}b^{\left(I\right)}} & J_{b^{\left(R\right)}t_{0}}\\ J_{b^{\left(I\right)}b^{\left(R\right)}} & J_{b^{\left(I\right)}b^{\left(I\right)}} & J_{b^{\left(I\right)}t_{0}}\\ J_{t_{0}b^{\left(R\right)}} & J_{t_{0}b^{\left(I\right)}} & J_{t_{0}t_{0}} \end{array}\right]}^{-1}{\left[\begin{array}{ccc} J_{pb^{\left(R\right)}} & J_{pb^{\left(I\right)}} & J_{pt_{0}} \end{array}\right]}^{T}. \end{array}$$

This concludes the derivation.
